# Osseous Metaplasia and Bone Marrow Elements in a Case of Renal Cell Carcinoma

**DOI:** 10.1155/2012/649257

**Published:** 2012-10-17

**Authors:** Seyma Ozkanli, Asif Yildirim, Ebru Zemheri, Sarp Korcan Keskin, Erem Kaan Basok

**Affiliations:** ^1^Departmant of Pathology, Goztepe Training and Research Hospital, SB Istanbul Medeniyet University, Doktor Erkin Caddesi, Kadıkoy, 34722 Istanbul, Turkey; ^2^Departmant of Urology, Goztepe Training and Research Hospital, SB Istanbul Medeniyet University, Doktor Erkin Caddesi, Kadıkoy, 34722 Istanbul, Turkey

## Abstract

Renal cell carcinoma with osseous metaplasia and bone marrow elements is a relatively rare event in these tumors. We discuss pathological differential diagnosis for this tumor with a review of the literature on this unusual case.

## 1. Introduction

Whereas focal calcificationsmay be present in renal cell carcinomas (RCC), metaplastic bone formation is a rare finding in RCC. We report a unique case of a large calcified renal cell carcinoma with massive osseous metaplasia and bone marrow elements.

## 2. Case Report

**Figure 1:**
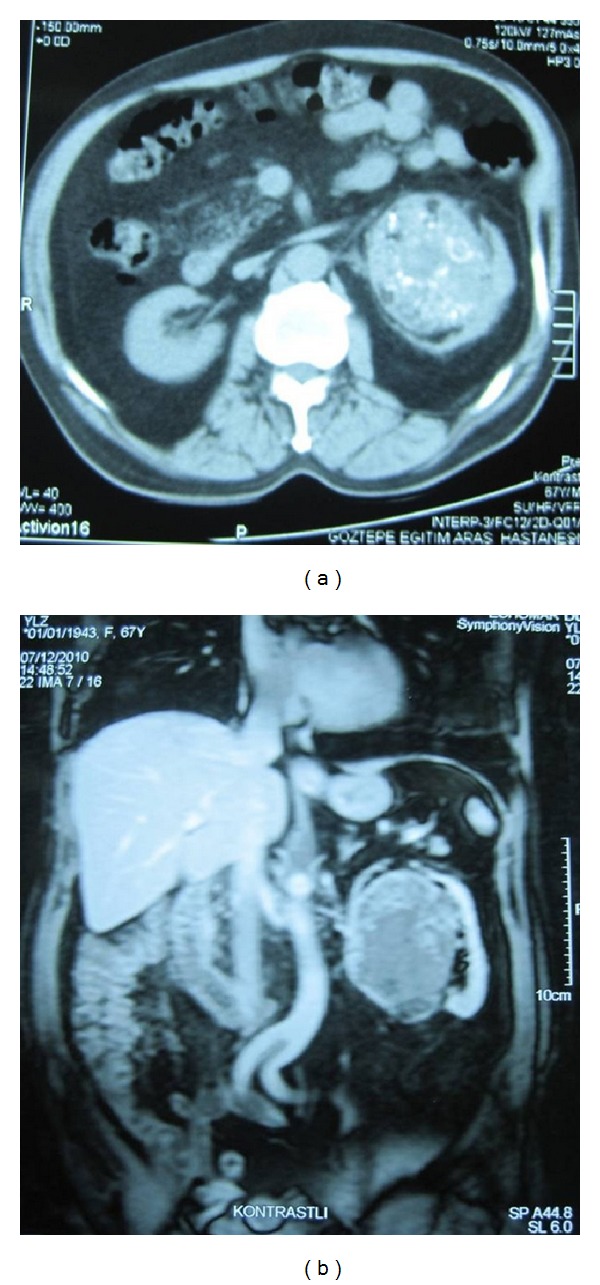
(a) Unenhanced CT scan of left kidney, showing areas of calcifications corresponding to metaplastic bone. (b) Coronal imaging of MR reveal a well-defined, solid tumor of left kidney with heterogenous enhancement.

A 68-year-old man was admitted with left lumbar pain. He was taking medications for coronary artery disease and chronic obstructive pulmonary disease and had no previous surgical procedures. The physical examination did not reveal an abdominal mass. There was no family history of kidney tumors. The complete blood count and differential were normal. The serum creatinine was 0.9 mg/dL (normal: 0.7–1.4 mg/dL) and liver function tests were normal. Abdominal ultrasonograhy and computerized tomography (CT) imaging revealed a round, sharply delineated, and diffusely calcified mass of 90 mm in diameter in the center of the left kidney (Figure 1). A left radical nephrectomy was performed without complications. The postoperative period was uneventful, and the patient was discharged 6 days after operation. 

**Figure 2:**
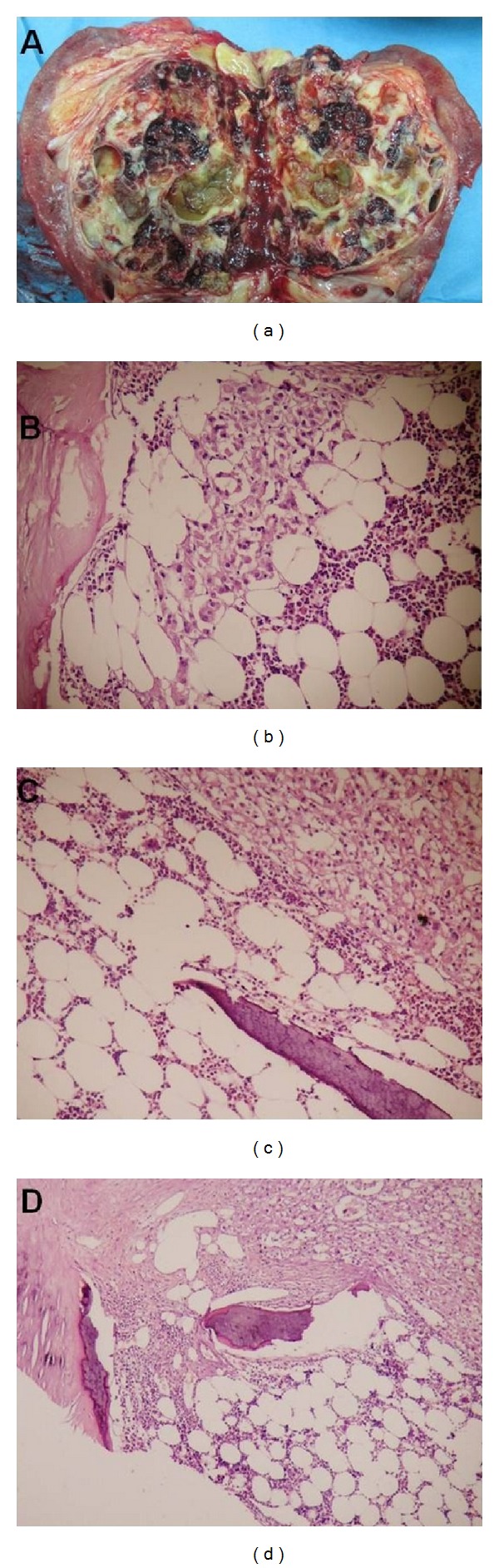
(a) Longitudinal section of specimen shows cystic lesions throughout the kidney. (b) Metaplastic bone intermingled with fat tissue containing myeloid and erythroid cells and varying numbers of foamy histiocytes (H&E x40). (c), (d) Lamellar bone forming a trabecula, adipose tissue, hemopoietic cells, and renal cell carcinoma (H&E x20).

Gross examination of the nephrectomy specimen revealed a 8.5 × 8 × 5 cm renal tumor. It is solitary well-circumscribed mass composed entirely of cysts, separated from adjacent renal parenchyma by a fibrous wall. The cysts containing hemorrhagic fluid are 1-2 cm in size. The cut section showed an extensively ossified mass that contained hemorrhagic, friable tissue with fat (Figure 2(a)). Histopathological examination of the tumor consisted of cysts separated by delicate septa, solid, trabecular sheets, and nesting of polygonal epithelial cells with abundant cytoplasm and prominent cell border. Besides, this tumor contained lamellar bone forming trabeculae intermingled with fat tissue containing myeloid and erythroid cells and varying numbers of foamy histiocytes (Figures 2(b)–2(d)). Thus, this case was reported as renal cell clear cell carcinoma with osseous metaplasia containing bone marrow. 

 The patient is alive and free from disease 18 months after the initial diagnosis. 

## 3. Discussion

Calcifications are present in a variety of renal lesions. Both benign and malignant neoplasms as well as benign cystic or inflammatory lesions may contain radiologically proven calcifications. Calcifications have been reported in RCC, angiomyolipoma, intrarenal aneurysms, cystic renal disease, renal abscess, echinococcal cysts, schistosomiasis, tuberculosis, xanthogranulomatous pyelonephritis, arteriovenous malformations, and hematoma [1]. Daniel et al. reported that 10.3% (58/560) of RCC had calcified foci through a radiologic review of 2709 renal masses at the Mayo Clinic [2]. Computerized tomography now demonstrates calcium in up to 31% of RCC, with an increasing incidence of calcifications noted with increasing size, as noted by Zagoria et al. [3]. Other malignant neoplasms of kidney, such as transitional cell carcinoma, Wilms tumor, and metastatic tumor may also contain calcification [2]. Because of its lack of vascularity and well-differentiated histology of calcified RCC, calcifications in RCC were once proposed to be a favorable prognostic factor [1–3]. The pattern of calcification in RCC may be curvilinear or punctuate [4]. The central calcification, regardless of pattern, can be a more reliable indicator of malignancy [2]. The rates of calcifications were 38% and 8–18% in chromophobe type and conventional type RCC, respectively. These results can support the indolent nature of the neoplasm and longer survival [5]. 

Osseous metaplasia in contrast to calcification is a rare event in any organ or tissue. Osseous metaplasia has been demonstrated in colorectal carcinomas, benign rectal polyps, malignant melanoma, breast carcinoma, hepatocellular carcinoma, fibrous histiocytoma, and perineuroma [6, 7]. It has been shown in the urothelial mucosa of the kidney related to pathogenesis of renal lithiasis. However, osseous metaplasia associated with RCC is exceedingly rare. In the exceptional major osteosarcoma of the kidney and in epithelial tumors, theoccurrence of osseous tissue, in association with renal tumors, has been documented [6].

The pathogenesis of ossification in tumors is unclear. A number of hypotheses have been suggested, including a metaplastic or reparative reaction to necrosis and degenerative changes in the tumor or peripheral tissues, and the production of bone by tumor cells [7]. Osseous metaplasia could be seen secondary to ischemia, necrosis, or inflammation in the tumor or surrounding tissue. In RCC, bone can originate through either the dedifferentiation of neoplastic cells into a sarcomatous proliferation (osteosarcomatous component), or the production of a dense collagenous matrix by mesenchymal cells with subsequent mineralization and organization into bone (osseous metaplasia) [8, 9]. This case belongs to this second category of epithelial tumors: bone tissue is adjacent to, while it does not arise from tumor cells, and it is associated with changes in tumor stroma rather than with the dedifferentiation of tumor cells. In addition, the fibroblasts of mesenchymal cell origin integrate the implant by forming collagen fibers and differentiating into osteoblasts and adipocytes which form the bony lamellae and bone marrow [8, 9].

Clinically in differential diagnosis, because of the extensive calcifications seen on CT scan and the long duration of the mass, several possibilities were mature cystic teratoma, renal cell carcinoma, adrenal neoplasm, soft tissue sarcoma, and metastatic carcinoma [10]. The histological appearance of the tumor directly ruled out this possibility.

In summary, we reported a unique case of renal cell carcinoma with the concomitant osseous metaplasia and bone marrow elements. 

## References

[1] Bloom TL, Gray Sears CL, Williams TR, Linfesty RL, Amling CL (2003). Multilocular cystic renal cell carcinoma with osseous metaplasia in a 25-year-old woman. *Urology*.

[2] Daniel WW, Hartman GW, Witten DM, Farrow GM, Kelalis PP (1972). Calcified renal masses: a review of ten years experience at the Mayo Clinic. *Radiology*.

[3] Zagoria RJ, Wolfman NT, Karstaedt N, Hinn GC, Dyer RB, Chen YM (1990). CT features of renal cell carcinoma with emphasis on relation to tumor size. *Investigative Radiology*.

[4] Weyman PJ, Mcclennan BL, Lee JKT, Stanley RJ (1982). CT of calcified renal masses. *The American Journal of Roentgenology*.

[5] Sharma S, Guglani B, Gamagati S (2004). Metastatic nodal calcification in aggressive chromophobe renal cell carcinoma. *Clinical Radiology Extra*.

[6] Fernandez-Conde M, Serrano S, Alcover J, Aaron JE (1996). Bone metaplasia of urothelial mucosa: an unusual biological phenomenon causing kidney stones. *Bone*.

[7] Cribbs RK, Ishaq M, Arnold M, O’Brien J, Lamb J, Frankel WL (1999). Renal cell carcinoma with massive osseous metaplasia and bone marrow elements. *Annals of Diagnostic Pathology*.

[8] Haddad FS, Shah IA, Manne RK, Costantino JM, Somsin AA (1993). Renal cell carcinoma insulated in the renal capsule with calcification and ossification. *Urologia Internationalis*.

[9] Garin JM, Marco I, Salva A, Serrano F, Bondia JM, Pacheco M (2007). CT and MRI in fat-containing papillary renal cell carcinoma. *The British Journal of Radiology*.

[10] Kefeli M, Yildiz L, Aydin O, Kandemir B, Faik Yilmaz A (2007). Chromophobe renal cell carcinoma with osseous metaplasia containing fatty bone marrow element: a case report. *Pathology Research and Practice*.

